# Evidence of Human Bourbon Virus Infections, North Carolina, USA

**DOI:** 10.3201/eid3011.240499

**Published:** 2024-11

**Authors:** Diana L. Zychowski, Gayan Bamunuarachchi, Scott P. Commins, Ross M. Boyce, Adrianus C.M. Boon

**Affiliations:** University of North Carolina at Chapel Hill, Chapel Hill, North Carolina, USA (D.L. Zychowski, S.P. Commins, R.M. Boyce); Washington University School of Medicine in St. Louis, St. Louis, Missouri, USA (G. Bamunuarachchi, A.C.M. Boon).

**Keywords:** Bourbon virus, ticks, tick-borne, vector-borne infections, neutralizing antibodies, viruses, seroprevalence, surveillance, North Carolina, United States

## Abstract

Bourbon virus is a tickborne virus that can cause human disease. Cases have been reported in Kansas, Oklahoma, and Missouri, USA. We identified Bourbon virus–specific neutralizing antibodies in patients from North Carolina. Bourbon virus infections are likely more common than previously thought, highlighting the need for improved diagnostics and surveillance.

Vectorborne diseases are a growing public health concern in the United States. Whereas bacterial pathogens are responsible for most infections, tickborne viruses represent an emerging and poorly understood threat (*1*).

Bourbon virus (BRBV), a tickborne virus belonging to the family Orthomyxoviridae, was first isolated from a patient living in Bourbon County, Kansas, USA, in 2014 (*2*). To date, human cases have been reported only in the United States, with 5 cases reported in 3 states: Kansas, Oklahoma, and Missouri (*2*–*5*). However, serosurveillance of St. Louis, Missouri, residents identified BRBV-specific serum-neutralizing antibodies in 0.7% (3/440) of persons, suggesting that BRBV infections are likely underrecognized (*6*).

Because of the scarcity of confirmed cases, descriptions of the clinical disease spectrum are limited. Fever, arthralgia, diarrhea, headache, and rash are complaints recorded early in the course of infection, followed by progression to critical illness in some cases. Laboratory abnormalities include leukopenia, thrombocytopenia, and elevated levels of aspartate and alanine aminotransferase (*2*,*3*,*7*).

The primary vector of BRBV is the lone star tick (*Amblyomma americanum*), which is widely distributed throughout the central, eastern, southeastern, and south-central United States (*4*,*8*). Although no cases of human BRBV disease have been confirmed in North Carolina, BRBV was isolated from ticks (North Carolina Division of Public Health, pers. comm., email, 2022 Jul 13), and neutralizing antibodies were detected in white-tailed deer across the state (Figure 1) (*9*). BRBV may be circulating in North Carolina and being transmitted to humans, possibly causing disease, but is undiagnosed or interpreted as other tickborne diseases. We used previously collected human serum samples to screen for the presence of BRBV-neutralizing antibodies.

**Figure 1:**
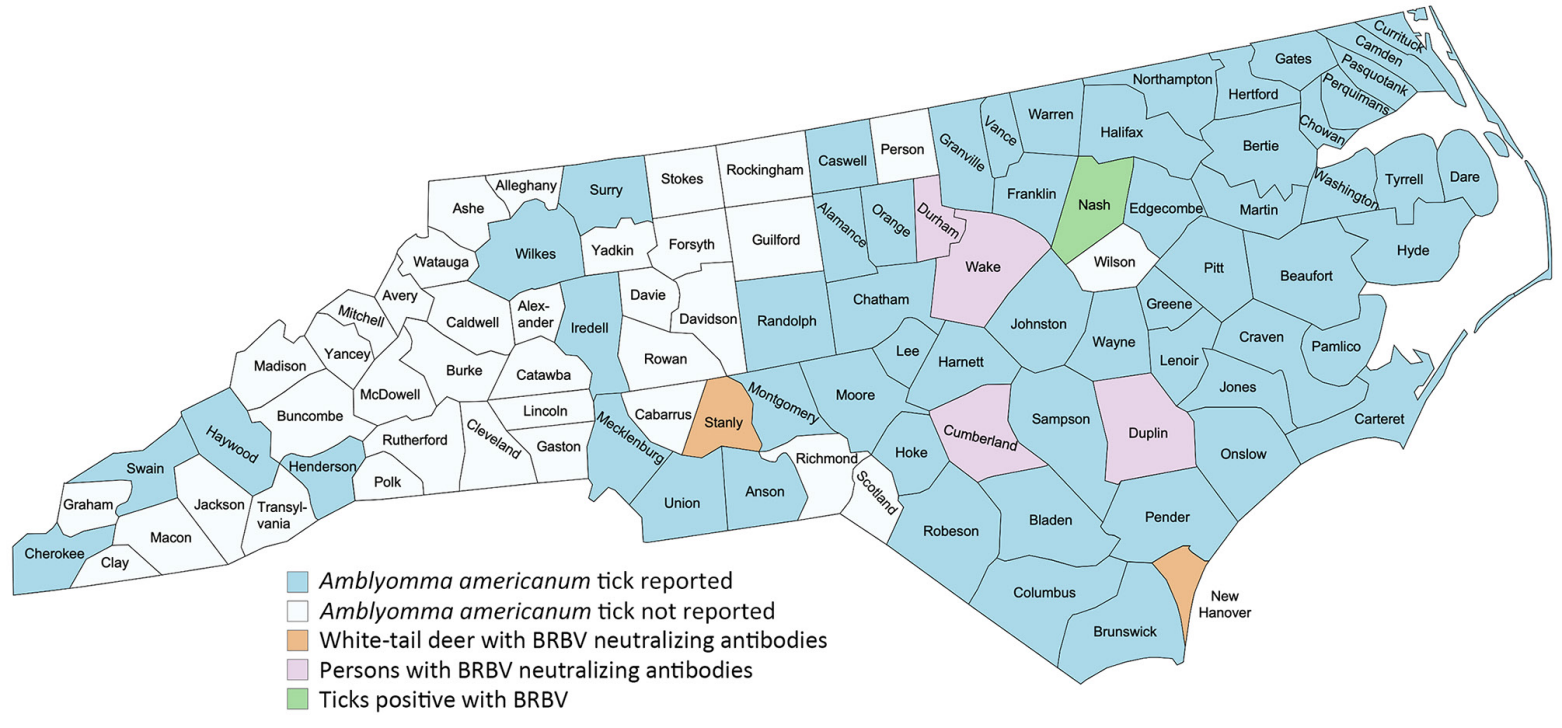
Distribution of the lone star tick (*Amblyomma americanum*) and evidence of Bourbon virus detection throughout North Carolina, USA. The map is adapted from a previous publication (*8*) and updated with locations of known bourbon virus carrying ticks (North Carolina Division of Public Health, pers. comm., email, 2022 Jul 13), white-tailed deer with positive bourbon virus–specific neutralizing antibodies (*9*), and persons with bourbon virus–specific neutralizing antibodies. Created by using http://www.mapchart.net.

## The Study

Serum from 518 residents of North Carolina, with variable known tick exposure, were screened for the presence of BRBV-neutralizing antibodies. Another 162 samples came from a cohort of patients with confirmed alpha-gal syndrome (AGS), a delayed-onset reaction following ingestion of mammal meat products that is associated with the bite of a lone star tick (*10*). An additional 156 samples were from a repository of recent heart valve recipients undergoing surveillance for the development of immunoglobulin E to galactose-α-1,3-galactose. The remaining 200 samples were from antenatal women. Those sample sources were selected because of availability, with the AGS group having the highest risk for tick exposure. Samples were collected during 2021–2023 and tested for BRBV antibodies in 2023.

We conducted testing by using previously validated methods (*6*). We diluted the serum samples 1:60 and screened in a rapid neutralization assay with a chimeric vesicular stomatitis virus (VSV) expressing the BRBV envelope protein to assess for the presence of BRBV-specific neutralizing antibodies. We conducted confirmatory testing on samples with >90% inhibition of VSV-BRBV by using a focus reduction neutralization test (FRNT) that used a BRBV St. Louis strain. We serially diluted serum samples 3-fold and incubated them with BRBV to reach a final serum dilution of 1:40–1:29,160 in media. We tested samples in duplicate. Each assay included convalescent serum from BRBV-infected mice as positive controls and samples without serum as negative controls. We calculated inhibitory values at a concentration of 90% (IC_90_) by using log (agonist) versus response. We defined samples with BRBV-neutralizing antibodies of IC_90_ >1:40 as positive and undertook a 10-year chart review of outpatient and inpatient records. The collection of the data used in the study was approved by the institutional review boards of Duke University (Durham, NC, USA) and the University of North Carolina, Chapel Hill (Chapel Hill, NC, USA).

**Figure 2:**
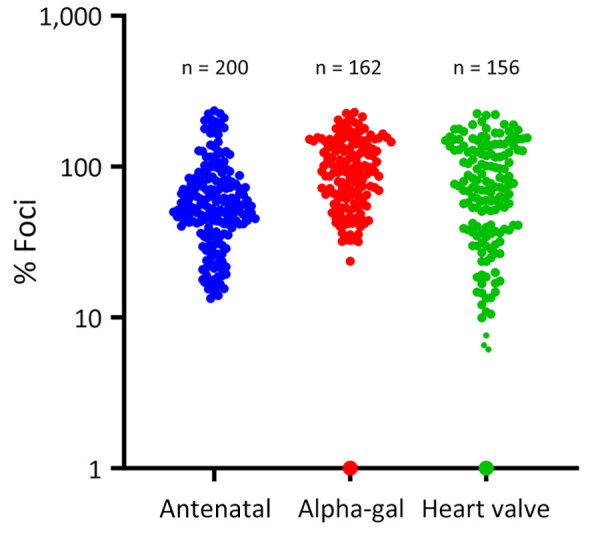
Results from a vesicular stomatitis virus and Bourbon virus rapid neutralization assay using serum samples from residents of North Carolina, USA. Normalized vesicular stomatitis virus-Bourbon virus neutralization (percentage foci compared with the control without serum) results are shown from 3 groups: antenatal women, persons with alpha-gal syndrome, and recent heart valve recipients. Colored dots represent singular serum sample.

Of the 518 samples, 6 (1.15%) demonstrated >90% inhibition of infection (Figure 2) in the VSV-BRBV rapid assay and underwent further testing by using BRBV FRNT. From the AGS cohort samples, 1 sample was positive for BRBV neutralizing antibodies, and from the heart valve group, 3 samples were positive for BRBV neutralizing antibodies. The IC_90_ for those 4 serum samples ranged from 1:100 to 1:200.

Among the 4 positive samples, all were residents of North Carolina (Table). Of those residents, 3 sought care for nonspecific viral respiratory symptoms within the previous 10 years, but there was no suspicion for a febrile tickborne illness. Only 1 person, with known AGS, had a documented history of tick bites.

**Table:** Documented characteristics of patients with bourbon virus–specific neutralizing antibodies, North Carolina, USA*

Case	Group	Age, y	County	Recent travel?	Tickborne illness?	Tick bite history?	Outdoor exposures	Comorbidities	Medical history
1	Heart valve	77	Cumberland	No	No	No	Outdoor walks	AS, essential thrombocytopenia, prior nephrectomy, OSA, HTN	Received care for allergies, possible viral upper respiratory infection, spring 2022.
2	Heart valve	78	Durham	No	No	No	None	AS, DMII, CKD, HTN, HLD, MGUS, ILD	Received antibiotics for possible lower respiratory infection superimposed on ILD, spring 2023.
3	Heart valve	79	Wake	No	No	No	None	AS, CAD, childhood rheumatic fever, HTN, HLD, hypothyroidism	Received care for respiratory viral infection or possible conjunctivitis, 2014.
4	AGS	63	Duplin	Arkansas, Montana	No	Yes, many	None	HTN, nephrolithiasis	Received multiple empiric doxycycline courses.

*AGS, alpha-gal syndrome; AS, aortic stenosis; CAD, coronary artery disease; CKD, chronic kidney disease; DMII, type two diabetes mellitus; HLD, hyperlipidemia; HTN, hypertension; ILD, interstitial lung disease; MGUS, monoclonal gammopathy of undetermined significance; OSA, obstructive sleep apnea.

## Conclusions

The presence of BRBV-specific neutralizing antibodies in 4/518 (0.77%) persons provides evidence of human BRBV infection in North Carolina. Whereas some of the patients did seek care for respiratory infections, there was no documented history of illness compatible with previous descriptions of BRBV infection. This finding suggests that there are likely to be asymptomatic or more subtle manifestations of BRBV infection, particularly in immunocompetent hosts, which would be consistent with other arboviral infections. Overall, our findings suggest that BRBV may be an underappreciated cause of vectorborne disease in NC.

Our study likely underestimates the number of persons infected with BRBV in North Carolina. It is possible we missed exposed persons who have waning immunity from more remote infections, or the infection did not induce sufficiently high levels of neutralizing antibodies to be detected in our assays.

The first limitation of our study is that the presence of neutralizing antibodies does not equate to clinical disease. This study aimed to demonstrate the presence of human infection in North Carolina but was not designed to capture a history of typical symptoms or risk factors for disease severity. Second, we cannot exclude travel of persons positive for BRBV neutralizing antibody to states with known cases. However, given the evidence of BRBV in North Carolina wildlife and ticks (*9*), it is not surprising that local BRBV transmission to humans has occurred. Finally, whereas we found a higher proportion of persons with BRBV-specific neutralizing antibodies within the heart valve group compared with those with known AGS, this study was not designed to look for statistical differences between groups and is subject to small frequencies.

The laboratory diagnosis of BRBV infection is challenging because there are no commercially available tests within the United States. Samples can be sent for serologic and nucleic acid amplification testing at public health laboratories or FRNT at the Centers for Disease Control and Prevention. Unfortunately, those tests have limitations. The viremic window for nucleic acid detection may be short or limited to the asymptomatic phase, and antibodies may not be detectable until 1 week after symptom onset. New diagnostic approaches are needed to improve accessibility and time to diagnosis, which cannot only prevent further invasive testing and unnecessary antimicrobial exposure but can also provide anticipatory guidance.

In addition to improved diagnostics, clinicians must remain vigilant to identify patients in need of viral testing. A single, acute serologic titer result cannot be used for the diagnosis of tickborne *Rickettsia* or *Ehrlichia* infections*.* For example, a North Carolina seroprevalence study revealed high population point prevalence rates for *Ehrlichia* infection of 8.6% (95% CI 5.9%–11.3%) and *Rickettsia* infection of 17.1% (95% CI 12.6–21.5) (*11*). Therefore, a single positive bacterial antibody titer should not preclude further tickborne diagnostic workup, particularly in cases of severe disease or where the patient fails to respond to antimicrobials.

Because of the clinical manifestation of nonspecific viral symptoms, challenging laboratory diagnostics, and the lack of commercially available tests, the true incidence and clinical symptomatology of BRBV remain unknown and active surveillance for acute cases is needed. Our findings substantially expand the known geographic area at risk for this emerging virus and demonstrate the need for further investigation and more widespread testing in patients with suspected BRBV infection.
